# Rare correlation of somatic *PRKACA* mutations with pregnancy-associated aldosterone- and cortisol-producing adenomas: a case report and literature review

**DOI:** 10.1186/s12902-024-01645-x

**Published:** 2024-07-16

**Authors:** Jianfan Lin, Yufei Li, Zhenxing Huang, Yingli Zhu, Li Li, Haiyan Yang, Xinghuan Liang, Yingfen Qin, Jia Zhou, Jing Xian, Deyun Liu, Decheng Lu, Zuojie Luo

**Affiliations:** 1 Department of Endocrinology, The First Affiliated Hospital of Guangxi Medical University, No.6, Shuangyong Road, Nanning, 530021 P.R. China; 2 Department of Urology, The First Affiliated Hospital of Guangxi Medical University, Nanning, 530021 P. R. China

**Keywords:** Adrenal adenoma, *PRKACA*, Co-secretion, Pregnancy, Somatic mutations

## Abstract

**Background:**

Somatic mutations have been observed to induce aldosterone-producing adenomas (APAs). These may be accelerated during pregnancy. Somatic PRKACA mutations are common in cortisol-producing adenomas (CPAs). However, their role in APAs, particularly aldosterone- and cortisol-producing adenomas (A/CPAs), is not well understood. This study aims to investigate the association between *PRKACA* mutations and the accelerated development of A/CPAs during pregnancy.

**Case presentation:**

A patient with primary aldosteronism (PA) associated with severe Cushing’s syndrome (CS) underwent surgical resection of an adrenal tumor one year after delivery. Pathologic examination revealed an adrenocortical adenoma characterized primarily by zona glomerulosa hyperplasia. Somatic mutation analysis revealed the presence of the somatic *PRKACA* mutation, which was validated as a deleterious mutation by various computational databases. Immunohistochemical results showed positive staining for cytochrome P450 family 11 subfamily B member 1 (CYP11B1), cytochrome P450 family 11 subfamily B member 2 (CYP11B2), and luteinizing hormone/chorionic gonadotropin receptor (LHCGR). Our study included a review of 20 previously documented cases of aldosterone- and cortisol-producing adenomas (A/CPAs), two of which were concurrently positive for both CYP11B1 and CYP11B2, consistent with our findings.

**Conclusion:**

Somatic mutations in *PRKACA* may correlate with the upregulation of LHCGR, which synergistically drives the accelerated growth of co-secretion tumors during pregnancy, thereby exacerbating disease progression.

**Supplementary Information:**

The online version contains supplementary material available at 10.1186/s12902-024-01645-x.

## Background

Primary aldosteronism is the leading cause of secondary hypertension. Early diagnosis and appropriate treatment are crucial for preventing harmful cardiovascular outcomes [1]. Recent research has demonstrated the need to view PA through a multidimensional and dynamic lens, encompassing manifestations ranging from subclinical to conventional clinical stages. In addition, PA is not a one-size-fits-all entity; areas of aldosterone production within the adrenal glands can range from unifocal to multifocal or even diffuse, affecting one or both adrenal glands [2]. As our understanding of PA evolves, its typology continues to expand. An intriguing facet of this expansion is the recognition of aldosterone and cortisol co-secreting adrenal adenomas (A/CPA), which are characterized by the simultaneous, autonomous hypersecretion of two corticosteroid hormones - aldosterone and cortisol. These A/CPAs represent a potentially overlooked subtype within the field of PA [3].

Aberrant activation of the cyclic adenosine monophosphate-protein kinase A (cAMP-PKA) signaling pathway is a key factor in the pathogenesis of adenomas originating in the adrenal cortex and characterized by cortisol hypersecretion [4]. *PRKACA* is the gene encoding the catalytic subunit of PKA and has been repeatedly implicated in cortisol-producing adenomas (CPAs) [5]. Notably, *PRKACA* mutations are rare in aldosterone-producing adenomas (APAs), and their precise mechanistic role in APAs remains elusive [6].

The secretion of human chorionic gonadotropin (hCG) increases during pregnancy. Some studies have suggested an association between hCG levels and tumor development and prognosis [7]. However, the exact mechanism by which hCG influences adrenal adenomas remains unclear.

This study presents the case of an individual with A/CPA. This patient had a marked onset of CS during pregnancy, with symptoms persisting and worsening for one year postpartum. Interestingly, the pathology of the adrenal adenoma in her case was predominantly characterized by bulbous band hyperplasia, a feature more akin to features seen in PA. Based on this case, we conducted a comprehensive review of the existing literature relevant to A/CPA.

## Case presentation and literature review

A 29-year-old woman was admitted to our hospital with the following symptoms: “protruding eyes, purple striae on the skin, secondary amenorrhea and abdominal distention for more than a year”. She used to be physically fit, focused on maintaining her figure, and enjoyed regular exercise. During the first trimester of her pregnancy, she experienced low progesterone levels and persistent vaginal bleeding, which were treated with progesterone at a local hospital. Third month of pregnancy, she noticed the development of purple striae on her lower abdomen along with acne. These purple striae gradually spread to her chest, both armpits and thighs. She gave birth vaginally in her 8th month of pregnancy.

**Fig. 1:**
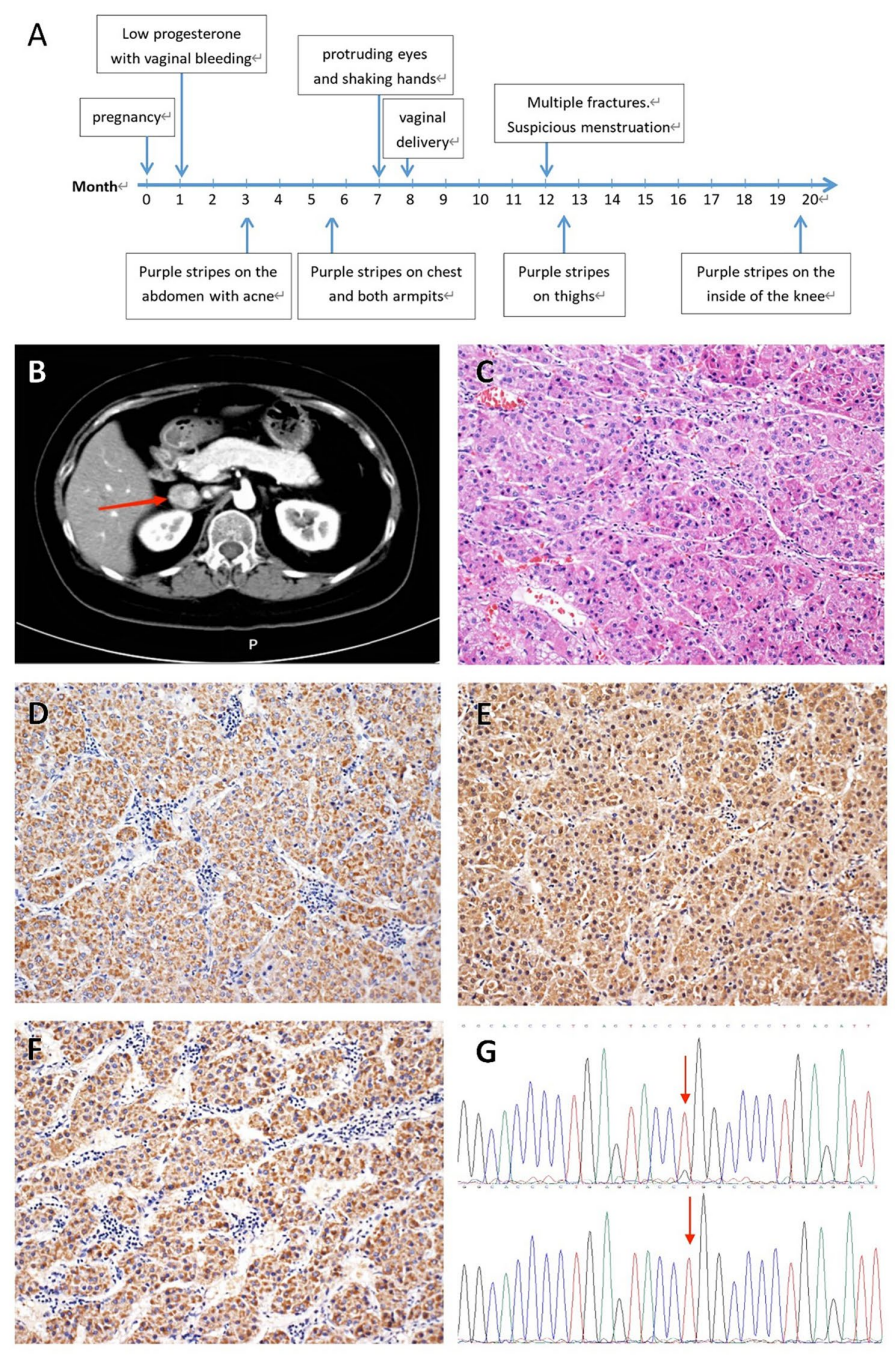
Patient Clinical Presentation. **(A)** Disease progression and symptoms onset in a patient; **(B)** Adrenal gland CT revealed a mass in the right adrenal region measuring 2.9 cm × 2.4 cm; **(C)** Hematoxylin and eosin staining of the tumor tissue; **(D-F)** Immunohistochemical staining of the tumor tissue, indicating positive expression of CYP11B1, CYP11B2, LHCGR; **(G)** Sanger sequence shows a partial change from T to G at base 617 of the coding region of *PRKACA*

In her fourth month postpartum, she had an accidental fall that resulted in multiple fractures of her right hip and ribs. As a result, she adjusted her postpartum recovery to focus on dietary management. While she gradually regained her pre-pregnancy weight, her limbs remained thin and hair growth increased. Her abdomen continued to be noticeably distended, and the purple striae on her skin worsened. One year after giving birth, these purple striae had spread to the inner knee joints on both sides (Fig. 1a). She sought treatment at our hospital’s endocrine clinic. The patient had only one irregular menstrual period after delivery, and the amount was significantly reduced compared to before her pregnancy. The patient denied any history of PA, CS, or related diseases, and she had no family history of these conditions.

She was admitted to the hospital for further evaluation. The cortisol rhythm was suggestive of cortisol dysregulation as shown in Table 1. High- and low- dose dexamethasone trials did not suppress cortisol levels, suggesting non-ACTH dependent cortisol elevation. These assay results are presented in Supplementary Material 1. The aldosterone-renin activity ratio (ARR) exceeded 30, indicating excessive autonomic aldosterone production. In the captopril suppression test, aldosterone levels were not suppressed, as shown in Table 2. 24-hour blood pressure fluctuated between 125–151/78–100 mmHg, with higher blood pressure readings during the night. Notably, systolic blood pressure did not follow the typical pattern of diurnal variation. Oral glucose tolerance tests (OGTT) suggested impaired glucose tolerance, while intravenous glucose tolerance tests (IVGTT) suggested hyperinsulinemia. In addition, high blood lipid levels were observed. A contrast-enhanced CT scan of the adrenal gland revealed a mass in the right adrenal region measuring 2.9 cm × 2.4 cm, with a mean density of 33 Hounsfield units (HU), consistent with a possible adenoma (Fig. 1b). Bone density was found to be lower than expected for the patient’s age. Dynamic contrast-enhanced MRI of the pituitary gland showed no clear abnormalities.

**Table 1:** Laboratory data

		before surgery	six months after surgery	reference point
PRA(ng/ml/h)	standing	0.43	3.31	0.93–6.56
	lying	0.22	1.94	0.05–0.79
ALD (pg/ml)	standing	106.9	127.4	74.8–345
	lying	92.2	111.6	57.5-201.3
AII(pg/ml)	standing	34.37	53.65	55.3-115.3
	lying	42.2	61.02	28.2–52.20
24-h UFC(nmol/24 h) (Urine volume)		4138.50 (2500 ml)	334.78 (1200 ml)	108–961 (1000-2000 ml)
Cortisol(nmol/L)	8AM	617.51	221.3	185–624
	4PM	546.14	49.73	107.8-333.6
ACTH(pg/ml)	8AM	3.31	52.32	7–65
	4PM	4.12	20.14	3–30
K+(mmol/L)		4.68	3.88	3.5–5.3

PRA, Plasma renin activity; ALD, Aldosterone; AII, Angiotensin II; ACTH, adrenocorticotropic hormone; 24-h UFC, 24-hour urinary free cortisol. Urinary cortisol was detected using chemiluminescence

**Table 2:** Captopril suppression test

	pre-dose	1 h post-dose	2 h post-dose
Cortisol (nmol/l)	581.43	552.82	546.52
AII (pg/ml)	60.86	46.19	57.1
ALD (pg/ml)	189.6	177.5	169.3
PRA (ng/ml/h)	0.32	0.28	0.36
ARR	59.25	63.39	47.03

Aldosterone levels were not suppressed. AII, Angiotensin II; ALD, Aldosterone; PRA, Plasma renin activity; ARR, aldosterone-renin activity ratio

We did not perform an adrenal venous sampling (AVS) because of the patient’s young age, significantly elevated aldosterone levels, and the presence of a unilateral adrenal tumor with features consistent with an adenoma. As a result, the patient was diagnosed with ACTH-independent CS and PA, both attributed to the adrenal adenoma, and was subsequently referred to the urology department for surgical intervention. Pathologic findings indicated the presence of a right adrenocortical adenoma. The tumor appeared on section as a gray-yellow to gray-brown solid mass characterized by predominantly zona glomerulosa hyperplasia with accompanying zona fasciculata and zona reticularis hyperplasia (Fig. 1c). Immunohistochemical results showed CD56 (+), Syn (+), CgA (-), Inhibin (+), CK (-), Vimentin (+), S-100 (-), and Ki-67# (+ 1%), supporting the above diagnosis. Silver staining and periodic acid-Schiff staining also supported this diagnosis. Because the patient’s Cushing’s symptoms manifested and worsened during pregnancy, additional immunohistochemistry was performed. The results were positive for CYP11B1, CYP11B2, and LHCGR (Fig. 1d-f).

After surgery, the patient received postoperative care, including symptomatic treatments such as gastric protection, pain management, and hormone hydrocortisone, calcium and potassium supplementation. During the six-month follow-up, there were significant improvements in the patient’s ocular edema, purpuric striae, and abdominal distension. Both cortisol and aldosterone levels improved significantly, as shown in Table 1. In addition, her blood pressure, blood lipid profile, blood glucose, and bone mineral density had returned to normal levels.

The somatic mutation analysis results suggested the presence of the *PRKACA* mutation (NM_002730: exon 7: c.T617G: p.L206R: rs386352352) in the patient. The mutation was subsequently confirmed through Sanger sequencing (Fig. 1G). Multiple computational tools, such as SIFT (which predicts deleterious effects with a score of 0.912), Polyphen2 (which predicts deleterious effects with a score of 0.899), PROVEAN (which predicts deleterious effects with a score of -5.16), and MutationTaster (which predicts deleterious effects with a score of 0.81), collectively suggest that the mutation is probably harmful or detrimental.

A literature review revealed that a total of 20 patients with A/CPA were reported between 2002 and 2022. Synthesizing the current cases, we observed a higher prevalence of female patients (76.2%). The age range varied widely, with no discernible pattern (ranging from 29 to 70 years). All patients had hypertension, but 23.8% did not have hypokalemia. Of note, 95% of patients had adrenal tumors larger than 2 cm in diameter. However, only 33.3% of the patients showed significant signs of CS. Among them, 3 patients tested positive for both CYP11B1 and CYP11B2 expression, representing 14.3% of the cases. It’s worth mentioning that most of the previous case reports did not analyze gene mutations, as shown in Table 3.

**Table 3:** Literature review

year	pmid	Case	gender	age	hypertension	hypopotassemia	pathologic changes in the adrenal glands	Tumor volume	specific immunohistochemistry	Mutation Gene	Obvious CS
2022	35,960,046	1	female	53	yes	yes	right adrenocortical adenoma	3 cm × 2.3 cm × 1.7 cm	CYP11B1(+), CYP11B2(+), CaSR(+), VD3R(+), PTH1R (+)	-	no
2022	35,960,046	2	male	59	yes	no	right adrenocortical adenoma	2.5 cm × 2.2 cm × 2 cm	CYP11B1(+), CYP11B2(+), CaSR(+), VD3R(+), PTH1R (+)	-	no
2022	35,707,125	1	female	49	yes	yes	right adrenocortical adenoma, light yellow	2 cm × 1.7 cm × 1 cm	-	-	no
2022	35,399,924	1	female	46	yes	yes	bilateral adrenal glands multiple aldosterone-producing micronodules(mAPM), aldosterone adenoma	1.6 cm(right), 2.1 cm(left)	CYP11B2(right, +), CYP11B1(left, +)	KCNJ5 (right side); PRKACA (left side)	no
2021	34,657,370	1	male	34	yes	yes	right adrenocortical adenoma	6.3 cm × 6 cm	CYP11B2(+), CYP11B1(-)	-	no
2021	34,384,396	1	female	67	yes	no	right Adrenal gland tumor	2.5 cm × 2.2 cm	CYP11B1(+), CYP11B2(-)	-	yes
2020	33,434,176	1	female	56	yes	yes	left adrenal gland tumor, golden-orange	4.5 cm × 3.1 cm × 3.2 cm	-	-	no
2016	8,533,534	1	female	50	yes	yes	right adrenal gland tumor, 2/3 golden yellow, 1/3 sepia	30 mm	-	-	yes
2014	25,228,847	1	female	29	yes	yes	left adrenocortical adenoma, golden yellow	2.1 cm × 1.6 cm	HSD3β1(+), P450-17A1(+)	-	no
2011	22,071,263	1	female	70	yes	yes	left adrenocortical adenoma, yellow	2 cm		-	no
2011	21,297,325	1	male	52	yes	yes	bilateral adrenal tumor	4.0 cm × 2.5 cm ×2.6 cm(left), 1 cm × 2.5 cm × 2 cm(right)	3β-HSD(+), P450C17(+)	-	no
2010	20,186,151	1	female	57	yes	yes	left adrenocortical adenoma	2.9 cm × 3.1 cm	CYP17(+)	-	yes
2010	20,186,151	2	female	49	yes	yes	right adrenocortical adenoma	3.5 cm	CYP17(+)	-	yes
2010	20,463,748	1	female	54	yes	no	right adrenal tumor, bilateral microAPA	2.5 cm(right), 2.1 mm(right), 1.6 mm(left), 1.9 mm(left)	3β-HSD(+), P450C17(+)	-	yes
2008	18,772,856	1	male	62	yes	yes	right adrenocortical adenoma, golden yellow	4.5 cm × 4.5 cm × 4.0 cm	-	-	no
2008	18,543,063	1	male	50	yes	yes	bilateral adrenocortical adenoma, golden yellow	1 cm × 1 cm(left), 1.1 cm × 0.8 cm (+)	3β-HSD(+), P450C17(+)	-	no
2007	17,541,216	1	female	35	yes	yes	left adrenocortical adenoma, golden-yellow	3 cm	P450c17(+)	-	no
2007	17,379,961	1	female	57	yes	no	right adrenal tumors, golden yellow with a few brown areas	2.1 cm, 1.9 cm	SCC(+), 3β-HSD II(+), CYP 21(+), CYP 11 B1(+), CYP 17(+)	-	no
2005	16,613,821	1	female	39	yes	no	left adrenocortical adenoma	3.0 cm X 2.5 cm	-	-	no
2002	12,487,169	1	female	43	yes	yes	right adrenocortical adenoma, golden yellow	4 cm	3β-HSD(+), P450 (aldo)(+), P450(11beta) (+)	-	yes
2023	-	1	female	29	yes	yes	right adrenocortical adenoma, grayish yellow-brown	2.9 cm × 2.4 cm	CYP11B1(+), CYP11B2(+), LHCGR(+)	PRKACA	yes

## Discussion

PA is a major contributor to secondary hypertension, which is characterized by autonomous secretion of aldosterone independent of renin and sodium levels. This leads to hyperactivation of the mineralocorticoid receptor (MR) [8]. In a cohort study, Markou et al. found that subclinical PA was prevalent in 13% of the normotensive population. Notably, these normotensive individuals had a more than 15-fold increased risk of developing hypertension within five years compared to those without PA [9].

Interestingly, a recent study using steroid hormone metabolomics identified cortisol co-secretion as a common factor in APA patients with metabolic complications such as type 2 diabetes mellitus, abnormal lipid metabolism, and osteoporosis [10]. This suggests that these complications may be more closely related to the effects of glucocorticoids than to excess aldosterone. In recent years, A/CPA has received increasing attention and is recognized as another subtype of adrenocortical adenoma that should not be overlooked. APAs typically have a diameter of less than 2 cm, while patients with A/CPA tend to have larger tumors and a higher incidence of cardiovascular complications, glucose intolerance/diabetes mellitus, and bone loss/osteoporosis [11].

CYP11B1 and CYP11B2 function as cortisol synthase and aldosterone synthase, respectively. In one particular study, CYP11B1 was found to be expressed exclusively in tumor tissue from a patient with PA but without significant CS. Conversely, CYP11B2 expression was restricted to the peritumoral area, which consisted of glomeruloid zonular cells [12]. Another study showed a correlation between increased A/CPA tumor volume and increased CYP11B1 expression, along with decreased CYP11B2 expression [13]. During our literature review, we also identified cases where both CYP11B1 and CYP11B2 were expressed in A/CPA tumors in some of our patients. This observation suggests that these two synthetic enzymes may play a critical role in the development of A/CPA.

Most benign adrenocortical lesions causing Cushing’s syndrome involve abnormalities in the cAMP or protein kinase pathways. For instance, inactivating mutations in the *PRKAR1A* gene, which encodes the regulatory subunit 1 alpha of protein kinase A, are linked to Carney complex [14]. One study identified CPA-related mutations in A/CPA, specifically involving *GNAS* and *PRKACA* [15], but the exact mechanism remains unclear. PKA is a tetramer composed of two catalytic (C) and two regulatory (R) subunits. The catalytic subunits exist in three isoforms, α, β, and γ, while the regulatory subunits have four isoforms: Iα, Iβ, IIα, and IIβ [16]. Previous clinical studies have consistently shown that somatic mutations in *PRKACA*, which encodes the catalytic subunit Cα, are predominantly associated with cortisol-producing adenomas exhibiting overt symptoms of CS [17]. However, in 2016, *PRKACA* somatic mutations were first identified in aldosterone-producing adenomas, and patients with p.Leu206Arg mutations presented with subclinical Cushing syndrome (SCS) [6]. It’s worth noting that the frequency of this mutation in aldosterone adenomas is relatively low.

The L206R mutation is the most common somatic mutation in *PRKACA*, in which a large, positively charged arginine (Arg) replaces leucine (Leu) [18]. This substitution creates a steric hindrance effect that can disrupt intermolecular interactions, chemical reactions, and the three-dimensional structure and function of biomolecules, including proteins. Consequently, this mutation interferes with holoenzyme assembly, resulting in constitutive PKA activation [17]. The affected amino acids are highly conserved and are located at the junction of the N and C lobes within the *P* + 1 loop of the C subunit. They play a critical role in creating hydrophobic pockets for substrate docking, substrate binding, recognition, and overall catalytic activity of PKA [19]. In the inactive state, each R subunit emits two inhibitory sequences that act as substrates or pseudosubstrates, effectively occupying the active site of the C subunit and preventing other substrates from entering. cAMP binding to the R subunits induces their dissociation from the C subunit, allowing the phosphorylation of numerous specific substrates [20]. The effect of the L206R mutation in *PRKACA* on PKA function is observed through four main pathways: (i) The L206R mutation eliminates binding between the R subunit and the C subunit, resulting in constitutive, cAMP-independent activation of PKA [21]; (ii) The L206R mutation induces RIIβ degradation by mediating cysteine-dependent Ser114 phosphorylation of RIIβ [22]; (iii) The L206R mutation alters the substrate preference of PKA and results in hyperphosphorylation of several PKA substrates [20]; (iv) The L206R mutation disrupts the hydrophobic interactions between the enzyme and the substrate, significantly reducing the binding affinity of the endogenous inhibitor PKI [19].

hCG belongs to the family of glycoprotein hormones and is a heterodimeric glycoprotein composed of two subunits: the α-subunit and the β-subunit. The α-subunit is shared with follicle-stimulating hormone (FSH) and luteinizing hormone (LH), while the β-subunit is specific for hCG [23]. LHCGR belongs to the family of G protein-coupled receptors, and its intracellular structural domains can bind to Gs proteins. It is distributed in the adrenal reticular and zona fasciculata [24]. Abnormal expression of steroidogenesis-related membrane hormone receptors in adrenocortical cells results in heightened sensitivity to hormones such as LH/hCG. These aberrant receptors can mimic the ACTH/cAMP signaling pathway, activating adenylate cyclase and converting ATP to cAMP, thereby promoting cortisol synthesis [25]. This mechanism elucidates how LH/hCG can stimulate cortisol production, leading to ACTH-independent macronodular adrenal hyperplasia and subsequently Cushing’s syndrome. When PKA is constitutively activated due to the *PRKACA* L206R mutation, the increase in cortisol synthesis becomes more pronounced during pregnancy. In addition, a study found increased LHCGR expression in APA [26]. In this study, LH treatment induced a concentration-dependent increase in CYP11B2 in H295R cells transfected with the LH receptor. It has been shown that CTNNB1 mutations can alter adrenal cell differentiation leading to overexpression of LHCGR and gonadotropin-releasing hormone receptor (GnRHR) in APAs [27]. A recent study showed that LHCGR overexpression in aldosterone adenomas arising during puberty, pregnancy or menopause with CTNNB1 mutations requires combined somatic mutations in *GNA11* and *GNAQ* [28]. LHCGR overexpression may also be associated with *PRKACA* somatic mutations, but further basic studies are needed to confirm this association.

Adrenal adenomas are typically benign tumors that grow slowly. Given the patient’s large tumor, it is plausible that an adrenal adenoma may have been present prior to pregnancy. However, the patient’s CS symptoms began to manifest and significantly worsen during pregnancy. We hypothesize that, on the one hand, the physiological increase in hCG during pregnancy may activate LHCGR in adrenal adenomas, thereby accelerating tumor growth and leading to increased synthesis and secretion of cortisol. On the other hand, the *PRKACA* mutation leads to constitutive activation of PKA, which further increases cortisol secretion. Both aldosterone and cortisol can bind to MR and has similar affinities for both hormones. The substantial amount of cortisol binding to MR results in patients with significant CS.

## Conclusion

In conclusion, we support the emerging concept of A/CPA as a distinct subtype of PA. Furthermore, our findings suggest a potential correlation between *PRKACA* somatic mutations and LHCGR overexpression, the combined effects of which may contribute to the rapid development of A/CPA during pregnancy and accelerate disease progression. However, the exact mechanisms underlying this interaction require further investigation. The overlap in symptoms between pregnancy and A/CPA presents a diagnostic challenge. In patients with suspected CS or PA symptoms during pregnancy, we recommend that appropriate biochemical tests and adrenal ultrasound be used in combination for early diagnosis. This approach helps avoid delays in treatment and minimizes potential harm to the pregnant woman and fetus.

### Electronic supplementary material

Below is the link to the electronic supplementary material.

Supplementary Material 1

## Data Availability

We will be able to provide only de-identified data of our patient to confirm/reproduce analysis. These could be requested by sending an email to the corresponding author Dr.Luo.

## Abbreviations

WES: whole-exome sequencing
CS: Cushing’s syndrome
PA: primary aldosteronism
LHCGR: luteinizing hormone/chorionic gonadotropin receptor
hCG: human chorionic gonadotropin
CYP11B1: cytochrome P450 family 11 subfamily B member 1
CYP11B2: cytochrome P450 family 11 subfamily B member 2
cAMP: cyclic adenosine monophosphate
PKA: protein kinase A
