# Supplementary material for: Rare correlation of somatic PRKACA mutations with pregnancy-associated aldosterone- and cortisol-producing adenomas: a case report and literature review
Source: BMC Endocr Disord. 2024 Jul 16;24:116. doi: 10.1186/s12902-024-01645-x (PMC11251286; doi:10.1186/s12902-024-01645-x)
Supplement: Supplementary file 1 — Supplementary Material 1 [file 12902_2024_1645_MOESM1_ESM.docx]

| 2022 Jun | | |  | |  | | |  |  |
| --- | --- | --- | --- | --- | --- | --- | --- | --- | --- |
| Classical high-dose dexamethasone trial | | | | | | | |  |  |
|  | | | pre-dose | | post-dose | | |  |  |
| 24-hour urinary free cortisol (nmol/24h) | | | >4138.5 | | >2648.64 | | |  |  |
| Urine volume (ml) | | | 2500 | | 1600 | | |  |  |
| Urinary cortisol (nmol/l) | | | >1655.4 | | >1655.4 | | |  |  |
| Serum cortisol (nmol/l) | | | 617.51 | | 531.89 | | |  |  |
| Urinary cortisol was detected using chemiluminescence. | | | | | | | |  |  |
| 2022 Jun | | |  | |  | | |  |  |
| Classical low-dose dexamethasone trial | | | | | | | |  |  |
|  | | | pre-dose | | post-dose | | |  |  |
| 24-hour urinary free cortisol (nmol/24h) | | | >4138.5 | | >3310.8 | | |  |  |
| Urine volume (ml) | | | 2500 | | 2000 | | |  |  |
| Urinary cortisol (nmol/l) | | | >1655.4 | | >1655.4 | | |  |  |
| Serum cortisol (nmol/l) | | | 617.51 | | 529.07 | | |  |  |
| Urinary cortisol was detected using chemiluminescence. | | | | | | | |  |  |
| 2022 Jun | | |  | |  | | |  |  |
| Laboratory data | | |  | |  | | |  |  |
|  |  | Before Surgery | | Half year  after Surgery | | reference point |  |  |  |
| Renin(pg/ml) | standing | 1.01 | | 20.07 | | 2.8-28.5 |  |  |  |
|  | lying | 0.77 | | 12.05 | | 1.8-24.5 |  |  |  |
| ALD(pg/ml) | standing | 60.12 | | 153.55 | | 31-351 |  |  |  |
|  | lying | 59.99 | | 76.32 | | 29-240 |  |  |  |
| ALD/Renin | standing | 59 | | 8 | | 0-57 |  |  |  |
|  | lying | 78 | | 6 | | 0-57 |  |  |  |
